# SpEx: a German-language dataset of speech and executive function performance

**DOI:** 10.1038/s41598-024-58617-3

**Published:** 2024-04-24

**Authors:** Julia A. Camilleri, Julia Volkening, Stefan Heim, Lisa N. Mochalski, Hannah Neufeld, Natalie Schlothauer, Gianna Kuhles, Simon B. Eickhoff, Susanne Weis

**Affiliations:** 1 Institute of Neuroscience and Medicine (INM-7 Brain and Behaviour), Forschungszentrum Jülich, Wilhelm-Johnen-Str., 52428 Jülich, Germany; 2 Institute of Systems Neuroscience, Heinrich-Heine University, Moorenstr. 5, 40225 Düsseldorf, Germany; 3 PeakProfiling GmbH, Eschenallee 36, 14050 Berlin, Germany; 4 Institute of Neuroscience and Medicine (INM-1 Structural and Functional Organisation of the Brain), Forschungszentrum Jülich, Wilhelm-Johnen-Str., 52428 Jülich, Germany; 5 Department of Psychiatry, Psychotherapy and Psychosomatics, Medical Faculty, RWTH Aachen University, Pauwelsstraße 30, 52074 Aachen, Germany; 6 Department of Neurology, Medical Faculty, RWTH Aachen University, Pauwelsstraße 30, 52074 Aachen, Germany

**Keywords:** Psychology, Human behaviour

## Abstract

This work presents data from 148 German native speakers (20–55 years of age), who completed several speaking tasks, ranging from formal tests such as word production tests to more ecologically valid spontaneous tasks that were designed to mimic natural speech. This speech data is supplemented by performance measures on several standardised, computer-based executive functioning (EF) tests covering domains of working-memory, cognitive flexibility, inhibition, and attention. The speech and EF data are further complemented by a rich collection of demographic data that documents education level, family status, and physical and psychological well-being. Additionally, the dataset includes information of the participants’ hormone levels (cortisol, progesterone, oestradiol, and testosterone) at the time of testing. This dataset is thus a carefully curated, expansive collection of data that spans over different EF domains and includes both formal speaking tests as well as spontaneous speaking tasks, supplemented by valuable phenotypical information. This will thus provide the unique opportunity to perform a variety of analyses in the context of speech, EF, and inter-individual differences, and to our knowledge is the first of its kind in the German language. We refer to this dataset as SpEx since it combines speech and executive functioning data. Researchers interested in conducting exploratory or hypothesis-driven analyses in the field of individual differences in language and executive functioning, are encouraged to request access to this resource. Applicants will then be provided with an encrypted version of the data which can be downloaded.

## Introduction

Research in the field of executive functioning (EF) and speech has suggested a strong relationship between the two, with studies implying the former to be a basic requirement for the latter^1–5^. Consistent with this relationship, clinical studies have shown an association between executive function impairment and various communication disorders including aphasia and language pragmatic disturbances^6^. Such communication disorders have been shown to result in symptoms across different levels of language including, but not limited to, processes involving lexicon, semantics, syntax, phonology, and prosody^7^.

Although the presence of a general relationship between language and cognitive performance is known, further studies can help to improve the understanding of the nuances of speech and their specific relationship with different aspects of EF. In the clinical context such analyses can be potentially beneficial for the identification of speech biomarkers for specific psychiatric disorders^8–11^.

Understanding the relationship between EF and speech inherently relies on measuring performance of both domains in a standardised manner. Throughout the years, several neuropsychological tests have been designed, with the primary purpose of capturing different executive abilities. Traditionally, such tests have been performed using paper and pencil, making them time-consuming, while requiring verbal administration and interpretation by trained administrators. Furthermore, standard pen-and-paper tests rely on manual scoring which could introduce errors and may thus lack sensitivity and specificity. Recent technological advancements have brought about the development of computerised versions of these tests which include automated scoring while also increasing the ease of administration. Studies have demonstrated reliability of individual assessments when comparing the computerised tests to the pen-and-paper versions^12–14^. In the case of speech, the most popular tests that are used in both the research and the clinical setting are formal speaking tests such as word generation and picture naming tasks. Depending on the type of speaking task, different aspects of speech can be extracted and different symptoms that can indicate different diseases can be identified. These tasks can also vary in the degree of experimental control that is used. Very structured tasks, such as counting from 1 to 10 or listing weekdays^15^, can provide insights into motor speech functions including respiration and phonation, articulation, resonance or prosody and potentially indicate diseases such as Parkinson’s diseases^16^ or Ataxia^17^. Additionally, verbal fluency tasks have been used extensively to assess planning ability and cognitive flexibility in diseases, such as aphasia or dementia^18^. Such formal tests tend to be of a controlled experimental nature, allowing for an easy extraction of variables of interest, focusing mostly on number of correct responses or errors, and reaction times. However, within the last few years qualitative analyses and related objective parameters were shown to provide deeper insights into the complex involvement of cognitive processes^19,20^. Such parameters can be extracted from tasks that allow participants more freedom in the speech that they produce due to less experimental control. One such example is the picture description task, which is commonly used to gain insights into syntactic structure as well as pragmatic competencies^21–23^. While the picture description task is framed by the content of the specific picture, interview situations and open questions provide more varied content as well as insights into more complex aspects of speech. Here, lexical selection, syntactic complexity, pragmatic aspects as well as voice modulation can be investigated^24^. However, the analysis of more qualitative aspects of speech is known to require manual transcription of audio files including labelling of sentence structures. Thus, qualitative speech analysis is extremely time-consuming and not feasible in the clinical context^25^. Recent technological advancements in speech recognition and computer-aided speech feature extraction provide a solution for the objective transformation and complex analysis of speech signals, present in audio data using *Natural Language Processing*, which results in the quantification of the data into vectors that represent the information that is related to the speech attribute of interest^26^. Such methods permit the time-efficient extraction of a multitude of variables that go beyond the ones that were traditionally analysed, thus pushing the boundaries of what can be analysed in the context of speech. Moreover, these automated extraction techniques, in combination with multivariate data-driven analytical techniques, pose fewer limits on the data that can now be used for analysis, allowing the analysis of rich, complex, and ecologically valid data such as spontaneous speech.

Over the years, research investigating the relationship between cognition and speech has been dominated by studies that average data over a group of participants^27^, ultimately treating variability as “noise”. As a result, findings yielding from such univariate within-group analyses fail to account for the rich variability that exists across individuals and thus lack generalizability. Recent work has shown that accounting for inter-individual variability, rather than disregarding it, can provide valuable information to the field^28–31^. This has led studies to explore relationships between different speech features and EF while using an individual-difference approach^32–36^. However, despite the noticeable move towards individual-difference studies, their number is still limited when compared to group-level studies. One of the main reasons for this is that analyses that take individual differences into account naturally require a larger number of participants. This, combined with the aforementioned need for manual coding associated with speech production data, has confined previous studies to smaller sets of data which has in turn limited the analyses that could be performed. Another issue contributing to the limitations of studies in the field is that since such studies are usually language-specific they must rely on datasets that contain speech data in the language of interest. To our knowledge, the only publicly available and well-sized dataset containing both speech and cognitive data is in the Dutch language^37^.

Considering all the points mentioned above, the goal of this work was to generate a German language dataset that would allow a rich variety of analyses in the field of cognition and speech with the possibility of taking individual differences into account. To this aim, the dataset capitalises on both the rich variety of attributes that can be found in speech as well as the modern methods that allow the extraction of such attributes. The generated dataset consists of speech data from 148 German native speakers with an age range of 20–55, supplemented by performance measures on several standardised, computer-based EF tests. The speaking tasks performed in this study ranged from controlled tasks such as word generation and interference tasks, to less controlled spontaneous speaking tasks that were designed in a way that mimics a natural conversation. The speech and EF data are complemented by a rich collection of demographic data as well as hormone information. This dataset is thus a large and expansive collection of data that spans a large age-range, that will allow a variety of analyses in the context of speech, EF, and inter-individual differences, and to our knowledge is the first of its kind in the German language. Some studies have linked free verbal reports (stories) to experience in an extension of the commonly used self-report surveys, e.g.,^38^. It could prove highly interesting to combine such finding with the present data to gain further insights into how experience is translated into language.

The data were collected at the Forschungszentrum Jülich in Jülich, Germany, between January and September 2018 in the context of a large-scale project aimed at investigating the relationship between speech and executive functioning. For each of the tests we provide the raw data output and the speech recordings. This data descriptor comprehensively describes the acquisition and curation of the dataset including the individual tests, experimental procedures and the folder structure of the data. The *Data Records* section describes how this data can be accessed. Researchers interested in performing exploratory and/or hypothesis-driven analyses in the field of language and cognitive performance are invited to make use of the collection presented here.

The dataset is owned by the Institute of Neuroscience and Medicine (INM-7, Brain and Behaviour) at the Forschungszentrum Jülich.

## Methods

### Ethics statement

This study, including the acquisition and sharing of the data, was approved by the ethics committee of the Heinrich-Heine-University Düsseldorf (Study number: 6055R). All procedures in this study were performed in accordance with the declaration of Helsinki, including but not limited to obtaining informed consent forms from each participant before conducting experimental measurements and keeping all private information anonymised. Participants were also informed that they could quit the study at any time if they wished to do so. All participants whose data is included in the dataset provided consent for the sharing of the data.

### Participants

This dataset includes 148 healthy participants with an age range of 20–55 [mean age 37.2 ± 11.1; 53 males (mean age = 35.6 ± 10.7); 95 females (mean age = 38.61 ± 11.4)]. Eligible participants included native German speakers who had not acquired an additional language before starting school and had no neurological or psychiatric diagnoses. Early bilingual participants were excluded from the study. Information on the ethnic make-up of the sample was not collected. Participants had different levels of education (finished secondary school = 4; professional school/ job training = 45; finished high school with a university-entrance diploma = 43; university degree = 56). Recruitment took place in North Rhine-Westphalia (Germany) via social networks and the Forschungszentrum Jülich mailing list. Testing sessions took place at the Forschungszentrum Jülich and took a duration of 150–180 min depending on the time needed for instructions and the speed with which the participants performed the tests. A remuneration fee of €50 was paid.

### Procedures

**Figure 1:**
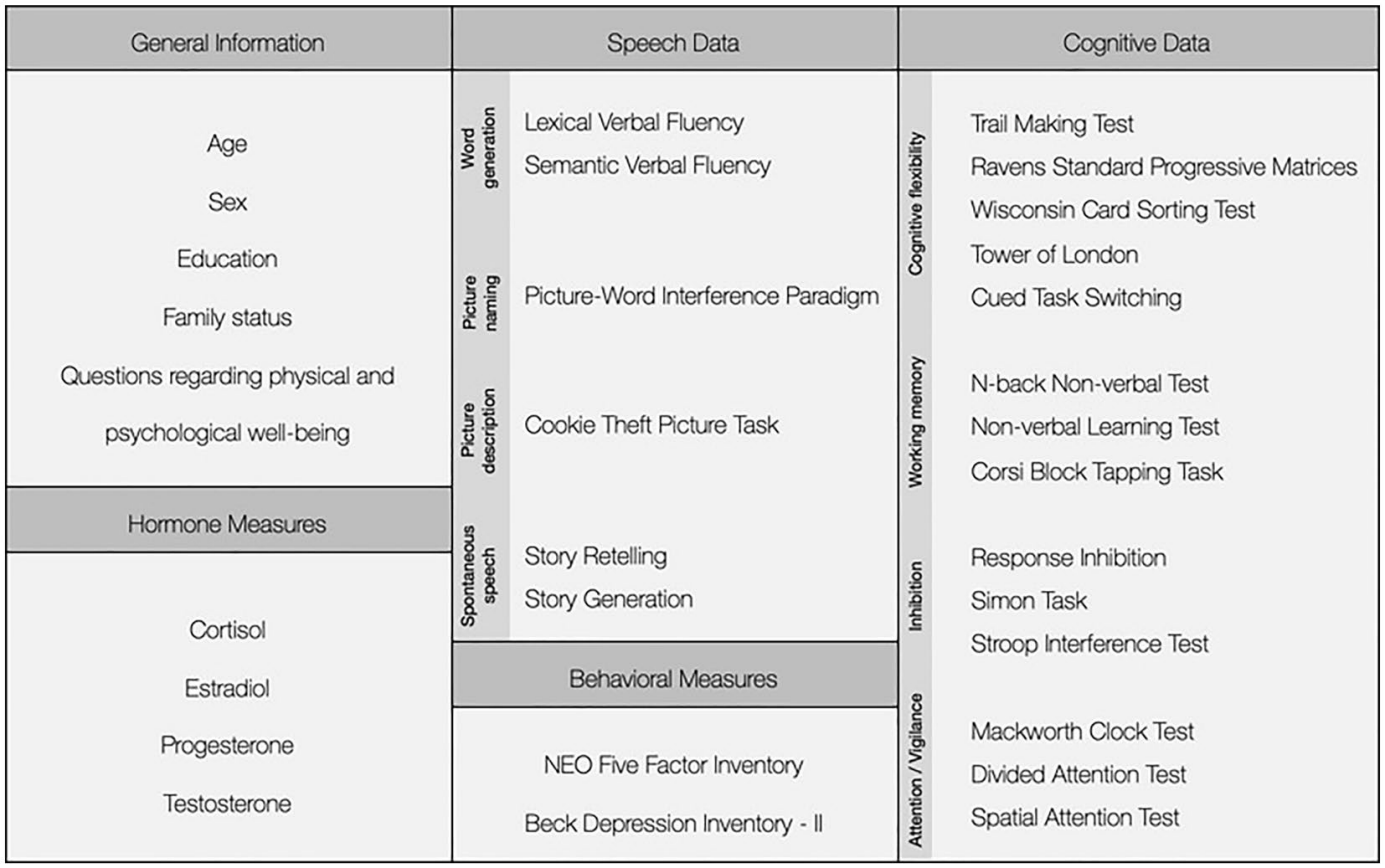
Summary of the measures that were acquired.

Data collection was performed by four examiners, all of whom were required to conduct several pilot tests and were instructed by the study leader to ensure a common standard. Each examiner gave standardised instructions before starting each test and help was provided by the examiner whenever the participant had any questions. The testing session included 4 speaking tasks and 14 EF tests. Acquired measures are provided in Fig. 1. Additional details for each of the tests are described below.

### Speaking test battery

The speaking test battery included in this study consisted of a number of well-known tools commonly used to test verbal abilities as well as spontaneous speaking tasks that were used to elicit discourse that is as close to natural speech as possible. When selecting the tests, care was taken to ensure that they cover a spectrum of tests that range from formal speaking tests such as word generation and picture naming to less structured tests such as picture description and spontaneous speech. All speaking tasks were presented and automatically recorded using Presentation software (Neurobehavioral Systems, Inc.; Version 20.1, Build 12.04.17) on an HP ProBook 4730s and using a Logitech Stereo USB Headset as a microphone.

#### Verbal fluency (VF)

The VF tasks used in this study were based on the *Regensburger Wortflüssigkeitstest*^39^ which is equivalent to the English *Controlled Oral Word Association Test*^40^. The implementation used in this study comprised two types of VF: lexical VF, and semantic VF, each consisting of three separate sub-tasks. The lexical VF task consisted of two simple tasks where participants were required to generate as many German words as possible that start with the letters “M” and “K” respectively. The decision for the selection of the specific letters was based on the difficulty level associated with the search for words starting with the respective letter. While words with the initial letter “M” provide an abundant search space, the letter “K” represents a higher difficulty level due to less available words^39^. An additional, more demanding task involved a switching component where the participants were required to switch between words that start with the letter “G” and words that start with the letter “R”. Each of the three tasks were performed for two minutes. Participants were not allowed to use proper nouns or repeat words more than once. Words with the same root were considered to be the same word and were thus also not allowed. Additionally, a word was only considered as correct if it would be found in a German book or newspaper. The instruction was given in German and was as follows:

‘Bei dieser Aufgabe sollen Sie innerhalb von zwei Minuten möglichst viele verschiedene Wörter nennen, die mit dem Anfangsbuchstaben “M” beginnen. Dabei sollen Sie verschiedene Regeln beachten: Sie sollen nur Wörter nennen, die in einer deutschen Zeitung oder einem deutschen Buch verwendet werden könnten. Dabei sollen Sie keine Wörter mehrfach nennen. Die Wörter dürfen aber auch nicht mit dem gleichen Wortstamm beginnen, also “Müll, Mülleimer, Müllabfuhr, Mülltonne”/“Kerze, Kerzenschein, Kerzenständer, Kerzenlicht” gelten nur als ein Wort. Weiterhin dürfen Sie auch keine Eigennamen nennen, also “Miriam, Max, Madrid, Malta”/“Kerstin, Kurt, Köln, Kreta” gelten nicht. Bitte versuchen Sie, möglichst schnell viele verschiedene Wörter mit dem Anfangsbuchstaben “M/K” zu nennen.’^39^

The semantic VF task consisted of two simple tasks where the participants were required to name animals and jobs respectively. The third task involved a switching component where the participants were required to switch between naming fruit and sports. Each of the three tasks were performed for two minutes and the rules specified in the lexical VF task still applied. The instruction was given in German and was as follows:

‘Bei dieser Aufgabe sollen Sie innerhalb von zwei Minuten möglichst viele verschiedene Wörter aus der Kategorie “Tiere”/“Berufe” nennen. Dabei sollen Sie keine Tiere mehrfach nennen. Bitte versuchen Sie, möglichst schnell viele Tiere/Berufe zu nennen. ’^39^

#### Picture-word interference paradigm

Participants were shown 64 different pictures (obtained from^41^), each accompanied by a spoken word which was either semantically related or semantically unrelated to the picture shown. The pictures were shown for 4500 ms and were followed by a fixation cross that was shown for 3000 ms. The auditory stimuli were spoken by a 23-year-old German female. Participants were required to name the picture that was shown as quickly as possible, and their audio was recorded as soon as the picture faded in. The list of picture names and auditory distractors can be found in the data repository as a separate file. For target and feature selection, items were controlled for an unequal onset and distractor items were not used as target items. Moreover, items were controlled for frequency and semantic relatedness using *GermaNet Pathfinder*^42^.

The instruction was given in German and was as follows:

“In dieser Aufgabe benennen Sie bitte wieder die Bilder, die Sie sehen. Während Sie das Bild sehen, werden Sie gleichzeitig Wörter hören. Diese Wörter können manchmal helfen, das Bild zu benennen oder sie erschweren es. Versuchen Sie trotzdem, das Bild so schnell es geht zu benennen.”

#### Picture description task

Participants were shown the *Cookie Theft Picture* obtained from^43^, and asked to describe it in as much detail as possible in 90 s. The instruction was given in German and was as follows:

“Bitte beschreiben Sie in 90 Sekunden dieses Bild so ausführlich wie möglich.”

The answer given by the participants was then recorded for 90 s.

#### Spontaneous speaking task

Participants were first told that they will be asked two questions to which they were required to reply in as much detail as possible for 5 min. This instruction was given in German and was as follows:

“Ich werde Ihnen heute insgesamt zwei Fragen stellen, bei denen ich Sie bitte, etwas ausführlicher für ca. 5 min zu antworten.“

The first question required the participants to either describe a book that they have read recently or to talk about something that they watched on television the night before. In case participants could not respond to this, they were asked to report any events happening within the last weeks. The question was asked in German and was as follows:

“Was haben Sie gestern Abend im Fernsehen geschaut oder welches Buch haben Sie gelesen? “

The second question required the participants to describe a vacation that they would like to take if time and money were no object. The question was asked in German and was as follows:

“Wo und wie würden Sie Ihren schönsten Urlaub verbringen? Erzählen Sie uns etwas darüber.“

Both answers given were recorded for 5 min each and the examiners asked for more detail in the case of participants that did not talk for that long.

### EF test battery

The EF test battery consisted of computerised versions of commonly used neuropsychological tests covering different subdomains of EFs either from the SCHUHFRIED Wiener Testsystem or Psytoolkit (https://www.psytoolkit.org/experiment-library/mackworth.html;^44,45^). The tests included in the battery were chosen to capture a broad range of subdomains of cognitive performance such as cognitive flexibility, planning, working memory, attention, and inhibition. There is overlap in the general areas covered by the tests. However, each test has properties that make it unique compared to the other tests in the battery. Table 1 outlines the specific battery and version that was used for each test while Table 2 provides an overview of the different variables that were measured for each test together with descriptive statistics for each of the variables (mean, standard deviation and range). Values being shared represent raw data for each test.

**Table 1:** A list of the EF tests performed by the participants.

Test	Battery	Form	Version	Testing time (minutes)
Corsi Block Tapping Test	*SCHUHFRIED Wiener Testsystem*	S1	28	10
Response Inhibition	*SCHUHFRIED Wiener Testsystem*	S1	23.04	10
Mackworth Clock Test	*PsyToolkit*	–	–	5
N-back Non-verbal Test	*SCHUHFRIED Wiener Testsystem*	S1	22.02	9
Non-verbal Learning Test	*SCHUHFRIED Wiener Testsystem*	S2	24.01	9
Simon Task	*PsyToolkit*	–	–	5
Raven's Standard Progressive Matrices	*SCHUHFRIED Wiener Testsystem*	S5	32.01	18
Stroop Interference Test	*SCHUHFRIED Wiener Testsystem*	S7	29	15
Cued Task Switching	*PsyToolkit*	–	–	5
Trail Making Test	*SCHUHFRIED Wiener Testsystem*	S1	51.2	3
Tower of London	*SCHUHFRIED Wiener Testsystem*	S11	23.04	11
Divided Attention	*SCHUHFRIED Wiener Testsystem*	S2	50.2	12
Spatial Attention & Neglect	*SCHUHFRIED Wiener Testsystem*	S1	23.05	12
Wisconsin Card Sorting Test	*PsyToolkit*	–	–	5

**Table 2:** An overview of the different variables measured for each test.

Test	Variable	Mean ± SD	Min to Max
Corsi block spanning test	Block span	5.64 ± 1.16	3 to 8
	Sum of correct items	10.50 ± 2.93	2 to 17
	Sum of false items	4.61 ± 1.37	3 to 9
	Sum of missed items	0.05 ± 0.21	0 to 1
	Sum of sequence errors	2.59 ± 1.31	0 to 6
Response inhibition	Reaction time^a^	0.53 ± 0.09	0.32 to 0.78
	Mean stop signal delay^a^	0.30 ± 0.07	0.05 to 0.35
	Stop signal reaction time^a^	0.22 ± 0.08	0.03 to 0 0.58
	Sum of commission errors	13.76 ± 6.33	1 to 31
	Sum of omission errors	1.33 ± 2.32	0 to 18
Mackworth clock test	Sum of missed jumps	8.17 ± 4.85	0 to 23
	Sum of false alarms	3.08 ± 3.80	0 to 28
N-back non-verbal test	Sum of correct items	8.27 ± 3.10	0 to 14
	Sum of commission errors	5.73 ± 3.10	0 to 14
	Sum of errors	8.45 ± 7.03	0 to 36
	Mean reaction time of correct items^a^	0.77 ± 0.19	0 to 1.36
	Mean reaction time of errors^a^	0.86 ± 0.25	0 to 1.64
Non-verbal learning test	Sum of correct responses	31.84 ± 4.91	11 to 39
	Sum of false responses	11.72 ± 8.35	0 to 48
	Sum of difference between correct and false responses	20.12 ± 7.75	− 13 to 35
	Processing time^a^	118.97 ± 33.14	74 to 303
Simon task	Sum of errors in congruent trials	1.01 ± 1.48	0 to 8
	Sum of errors in incongruent trials	3.16 ± 2.90	0 to 19
Ravens standard progressive matrices	Sum of correct items	27.73 ± 3.64	14 to 32
	Processing time^a^	638.74 ± 162.12	328 to 905
Stroop interference	Reading interference^a^	0.15 ± 0.09	− 0.04 to 0.40
	Naming interference^a^	0.13 ± 0.08	− 0.02 to 0.38
	Interference difference^a^	− 0.06 ± 0.12	− 0.40 to 0.23
	Number of false reactions (reading baseline)	2.14 ± 2.58	0 to 13
	Number of false reactions (naming baseline)	2.72 ± 2.68	0 to 18
	Number of false reactions (reading interference)	3.71 ± 8.06	0 to 96
	Number of false reactions (naming interference)	3.54 ± 3.18	0 to 23
	Processing time	405.84 ± 59.88	294 to 616
Cued task switching	Sum of errors	2.46 ± 3.13	0 to 19
	Sum of timeouts	0.29 ± 0.87	0 to 7
	Sum of errors in incongruent trials	2.02 ± 2.40	0 to 14
	Mean reaction time for incongruent trials^a^	0.6 ± 0.14	0.37 to 1.34
	Mean reaction time for congruent trials^a^	0.55 ± 0.13	0.31 to 1.11
	Switch costs (RT incongruent–RT congruent)^a^	0.05 ± 0.08	− 0.19 to 0.39
Trail making test	Processing time for part A^a^	17.44 ± 3.48	9.69 to 27.72
	Processing time for part B^a^	25.70 ± 8.58	12–24 to 57.55
	Difference part B–part A^a^	8.25 ± 7.14	− 3.32 to 40.57
	Quotient of B/A^a^	1.47 ± 0.39	0.84 to 3.39
	Sum of errors in part A	0.07 ± 0.25	0 to 1
	Sum of errors in part B	0.68 ± 1.09	0 to 9
Tower of London	Planning ability	7.47 ± 2.31	1 to 12
	Sum of correct responses	10.45 ± 1.80	2 to 12
	Sum of self-corrections	1.77 ± 2.09	0 to 11
	Sum of wrong pole choices	0.70 ± 2.76	0 to 31
	Sum of blocked pole choices	1.13 ± 2.73	0 to 22
	Sum of impossible position choices	0.81 ± 2.85	0 to 28
Divided attention	Sum of missed items (unimodal visual)	2.12 ± 3.19	0 to 16
	Sum of false alarms (unimodal visual)	3.24 ± 4.51	0 to 42
	Mean reaction time (unimodal visual)^b^	454.30 ± 97.29	251 to 826
	Sum of missed items (cross modal)	2.79 ± 3.13	0 to 17
	Sum of false alarms (cross modal)	3.26 ± 5.99	0 to 49
	Mean reaction time (cross modal)^b^	469.49 ± 111.57	240 to 880
Spatial attention	Mean reaction time of unannounced items^b^	384.53 ± 50.72	289 to 526
	Sum of missed items when correct item is announced	0.99 ± 1.48	0 to 8
	Mean reaction time when correct item is announced^b^	311.15 ± 50.88	207 to 494
	Sum of missed items when incorrect item is announced	0.17 ± 0.43	0 to 2
	Mean reaction time when incorrect item is announced^b^	345.75 ± 52.43	238 to 483
	Mean reaction time (short SOA)^b^	353.32 ± 47.09	261 to 485
	Mean reaction time (Long SOA)^b^	345.18 ± 46.58	256 to 474
	Sum of errors	3.27 ± 3.05	0 to 16
Wisconsin card sorting test	Sum of errors	12.83 ± 5.86	6 to 36
	Sum of perseveration errors	8.02 ± 3.23	4 to 21
	Sum of non-perseveration errors	4.81 ± 3.31	0 to 18
	Sum of timeouts	0.53 ± 1.05	0 to 9

SOA = stimulus onset asynchrony.

^a^Time measured in seconds.

^b^Time measured in milliseconds.

#### Corsi block tapping test (CORSI)

Participants were presented with nine cubes arranged in an irregular order on the screen followed by a pointer that points to three cubes in a specific order. At the end of this sequence a signal sounded prompting the participants to repeat the given sequence. The length of the sequence was increased by one cube each time the participants completed the sequence successfully.

#### Response inhibition (INHIB)

The test consisted of two parts. In the first part of the test an arrow was displayed on the screen and participants were asked to respond to the direction in which the arrow was pointing. In the second part of the test the participants were asked to repeat the task as in the previous part but were additionally asked to suppress their motoric response whenever they heard an auditory signal.

#### Mackworth clock test (MACK)

Participants were presented with a large green clock hand displayed on a black screen. The hand moved like the second hand of a clock, approximately every second. At infrequent and irregular intervals, the hand made irregular “jumps”. Participants were requested to detect and quickly react to these irregular “jumps” by pressing a button. The irregular “jump” of the clock hand was around 10% of the circle and the duration of the test was 1 min comprising 60 total moves of the clock hand.

#### N-back non-verbal test (NBN)

A sequence of 100 abstract figures were presented one by one. The task consisted of indicating whether the figure that was currently displayed was identical to the one shown two places back (2-back paradigm). If it was, the participant was expected to press a button as quickly as possible.

#### Non-verbal learning test (NVLT)

Nonsensical, irregular, and geometric figures were presented on the screen. During the course of the test some figures were shown multiple times. For each figure the participants were required to decide whether the current figure has already appeared or whether this figure is being shown for the first time.

#### Simon task (SIMON)

Participants were asked to press the “l” key if they read the word "rechts" (German word for *right*) and the “a” key if they read the word "links" (German word for *left*). Each word was displayed either on the right or the left part of the screen meaning that the stimulus could be congruent or incongruent to its position.

#### Ravens standard progressive matrices (SPM)

The participants were shown eight separate items that follow a pattern. The task required the participants to identify one missing item out of 6 choices to complete the pattern. The difficulty in pattern recognition increased during the course of the test.

#### Stroop interference test (STROOP)

Names of colours were displayed on the screen in a colour which was incongruent to the name (e.g., the word "blau" (German for *blue*) printed in red). The test consisted of two conditions. In the naming condition the participants were asked to respond to the colour of the words. In the reading condition participants were asked to respond to the meaning of the word. A baseline measure for the reaction speed and accuracy of the participants was established at the start of the test by presenting colour words without colouring or simple colour bars.

#### Cued task switching (SWITCH)

This task consisted of a shape and a colour task. A cue stimulus informed the participant which task to perform on every trial. The cue for the colour task was the word “COLOR” and the cure for the shape task was the word “SHAPE”. In the colour task participants were asked to respond to the colour of the presented figure while ignoring the shape. In the shape task participants were required to respond to the shape of the presented figure while ignoring the colour. For selecting the respective colour or shape, two letters of the keyboard were determined (the letter “b” was used for the answers *circle* and *yellow* and the letter “n” was used for the answers *rectangle* and *blue*. Depending on the answer, the respective letter was pressed by the participant.

#### Trail making test (TMT)

The task consisted of 2 parts: part A and part B. In part A numbers ranging from 1 to 25 were displayed randomly across the screen. Participants were asked to click on the numbers in ascending order and as quickly as possible. In part B, the numbers displayed on the screen ranged from 1 to 13 and were accompanied by alphabetic letters ranging from A to L, both of which were presented in a random order. Part B required participants to click on numbers and letters alternately and in ascending order.

#### Tower of London (TOL)

Participants were presented with an image that depicts a three-dimensional wooden model with three rods on which three balls of different colors are placed. The left rod holds three balls, the middle rod takes two balls, and the right rod has room for one ball. The participants were asked to move the balls from the starting state to a target position using a minimum number of moves. The target state was always shown in the upper part of the screen and the starting state in the lower part. The minimum number of moves required to achieve this was shown to the left of the starting state. Various rules were to be observed, one of which was the rule that only one ball can be moved at a time.

#### Perception and attention functions test: divided attention (WAFG)

The participants were required to focus on two geometric figures and one auditory stimulus. At certain intervals the stimuli change their intensity (i.e., figure gets lighter and/or auditory stimulus gets louder). The participants were asked to respond when two stimuli became lighter/louder twice in succession.

#### Perception and attention functions test: spatial attention & neglect (C)

Four triangles were presented in four spatial positions. The participants were required to react if a triangle changes intensity (i.e., gets darker). In the neglect test an interfering or matching visual cue was also given.

#### Wisconsin card sorting test (WCST)

The task used here is not the actual Wisconsin Card Sorting Test, as copyrighted in the US, but rather a computer-based task that is inspired by the original test^46^. Four stimulus cards illustrating different geometrical figures were presented. The figures on the cards differ in number, colour, and form. The task of the participants was to figure out the classification rule to be able to match a newly presented card to one of the four cards. Participants were given feedback for every card that they matched. The classification rule was changed every 10 cards, requiring the participants to shift rules accordingly.

### Additional data

In addition to the main set of speaking and EF tasks, phenotypical data were collected through questionnaires including the German version of the Beck Depression Inventory (BDI-II^47^) used to collect information regarding depressive symptoms, and the NEO Five Factor Inventory (NEO-FFI^48^). Furthermore, participants were asked general questions about their background, habits, and their physical and psychological well-being before commencement of the testing session. Saliva samples were collected at the beginning and at the end of the test session, stored in a refrigerator and sent to an external lab for analysis. The two saliva samples of each participant were then pooled at an external lab which carried out quantification analyses for cortisol, progesterone, oestradiol, and testosterone.

### Data records/usage notes

The dataset presented in this paper is stored on GDPR-compliant and protected servers of the Forschungszentrum Jülich, housed at the Jülich Super Computing Centre, as agreed upon by the participants. The dataset complies with the four basic principles of FAIR. The dataset is clearly described with metadata, that are accessible on Jülich DATA (https://data.fz-juelich.de/dataset.xhtml?persistentId=doi:10.26165/JUELICH-DATA/CHWZDZ) making it findable, accessible, interoperable and reusable.

Researchers who wish to acquire access to the data are kindly asked to contact the authors at spexdata@fz-juelich.de. Applicants will be asked to submit an approved ethics application together with a project outline. Additionally, applicants will be asked to ensure that the requested data will be only used for the research project specified and that it will not be passed on to third parties. Once the request is approved applicants will receive temporary access to an encrypted version of the data which they can then download.

The dataset repository contains 76.81 GB of data and includes five main folders, four of which contain the different measures depicted in Fig. 1 (i.e., EF data; speech data; hormone data; questionnaires). A fifth folder presents publications that have already made use of the dataset^32,33^. The folder containing the EF data contains a sub-folder for each of the different tests used (i.e., the 14 tests listed in Tables 1 and 2). Each sub-folder contains a comma-separated-value file with the corresponding raw data as well as a text file consisting of information about the specific measure, details on how it was acquired as well as details of the hardware and software used for the acquisition. On the other hand, the folder containing the speech data contains a sub-folder for each of the participants. Each of these sub-folders contains further sub-folders for each of the 6 speaking tasks, which in turn contain the corresponding raw waveform audio files in the uncompressed format RIFF WAVE (WAV). All the speech utterances were recorded with a bit rate of 2822 kBit/s, a sample size of 32 bit, and a sampling rate of 44.100 kHz.

For each of the 148 participants, 6 min of Lexical Verbal Fluency, 6 min of Semantic Verbal Fluency, 4.13 min of the Picture-Word Interference Paradigm, 1.5 min of the Picture Description Task, 5 min of Story Retelling, and 5 min of Story Generation were recorded. This corresponds to a total of 27.63 min of recorded speech data per subject. In total, the dataset provides 68.16 h of speech recordings.

We expect this dataset to be of interest to researchers conducting exploratory or hypothesis-driven research in the field of individual differences in language and executive functioning. The dataset has already been used to predict verbal fluency scores from EF performance^32^, and to predict EF performance from a comprehensive set of verbal fluency features^33^.

The data were collected at the Forschungszentrum Jülich in Jülich, Germany, between January and September 2018 in the context of a large-scale project aimed at investigating the relationship between speech and executive functioning. For each of the tests we provide the raw data output and the speech recordings. This data descriptor comprehensively describes the acquisition and curation of the dataset including the individual tests, experimental procedures and the folder structure of the data. The *Data Records* section describes how this data can be accessed. Researchers interested in performing exploratory and/or hypothesis-driven analyses in the field of language and cognitive performance are invited to make use of the collection presented here.

## Data Availability

The dataset presented here will be made available to interested researchers upon request, as described in this data descriptor. Researchers who wish to acquire access to the data are kindly asked to contact the authors at spexdata@fz-juelich.de.
